# Hepatic Lipidomics Unravels the Lipid‐Lowering and Anti‐Obesity Efficacy of Diacylglycerol Oil: Mechanistic Insights From High‐Fat Diet‐Induced Obese Mice

**DOI:** 10.1002/fsn3.70395

**Published:** 2025-06-13

**Authors:** Lina Shi, Yiran Liu, Yuanyuan Yan, Dongsheng Bian, Jun Jin, Qingzhe Jin, Jiai Yan, Xingguo Wang

**Affiliations:** ^1^ State Key Laboratory of Food Science and Resources, School of Food Science and Technology Jiangnan University Wuxi China; ^2^ Clinical Nutrition Department Affiliated Hospital of Jiangnan University Wuxi China; ^3^ School of Public Health Shanghai Jiao Tong University School of Medicine Shanghai China; ^4^ Department of Clinical Nutrition, Ruijin Hospital Shanghai Jiao Tong University School of Medicine Shanghai China

**Keywords:** anti‐obesity, diacylglycerol, lipid‐lowering, lipidomics

## Abstract

This study employed a multidimensional approach combining clinical and animal experiments to elucidate the lipid‐modulating mechanisms of diacylglycerol (DAG). In a 12‐week intervention involving obese individuals, fasting serum triglyceride levels were significantly reduced in the DAG group compared to baseline. Within‐group reductions in triglycerides and low‐density lipoprotein (LDL) cholesterol were more pronounced in the DAG group than in the triacylglycerol (TAG) control group (*p* < 0.05). In a high‐fat diet‐induced obese mouse model, DAG significantly lowered serum total cholesterol, LDL levels, visceral fat weight (*p* < 0.05), attenuated hepatic steatosis, and altered hepatic lipid distribution. Lipidomic profiling revealed that DAG markedly downregulated hepatic triglycerides, ceramides, and monoacylglycerols, while normalizing sterol lipid levels. Pathway analyses based on differential lipids showed that DAG affected hepatic lipid composition mainly by intervening in the glycerophospholipid metabolism pathway. Mechanistically, DAG suppressed the expression of stearoyl‐CoA desaturase 1 and fatty acid synthase, while upregulating carnitine palmitoyltransferase 1, thereby enhancing hepatic lipid metabolism through dual regulation: inhibition of synthesis and promotion of catabolism and oxidation. These findings reveal DAG's structure‐dependent role in restoring lipid homeostasis and provide a theoretical foundation for functional lipid‐based strategies targeting metabolic disorders.

## Graphical abstract

In a 12‐week clinical trial, DAG reduced serum TG and LDL cholesterol in obese individuals. In high‐fat diet mice, DAG lowered lipids, visceral fat, and hepatic steatosis, acting via inhibiting lipid synthesis (SCD1, FASN) and promoting fatty acid oxidation (CPT‐1) while modulating glycerophospholipid metabolism.

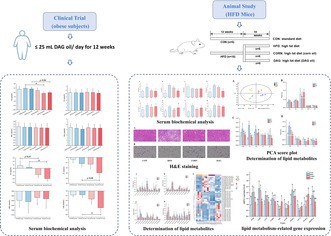

## 1 Introduction

Obesity as a pandemic‐scale metabolic syndrome with complex etiological underpinnings has reached pandemic proportions with steadily rising prevalence over five decades (Lin and Li 2021; Rössner 2002). Obesity is a critical risk amplifier for cardiovascular pathologies and all‐cause mortality, establishing itself as an enormous public health challenge (Van Gaal et al. 2006). The homeostatic regulation of energy expenditure and caloric intake constitutes a fundamental mechanism in weight management, with obesity pathogenesis fundamentally arising from chronic dysregulation of energy equilibrium between metabolic utilization and nutritional assimilation (Oussaada et al. 2019). Contemporary epidemiological research has demonstrated that modifications to dietary intake, particularly the increase in fat consumption, are a significant contributing factor to the prevalence of obesity (Buckley 2018). Chronic high‐fat dietary induces promote systemic triacylglycerol (TAG) deposition, thereby establishing a pathophysiological foundation for obesity development (Hill et al. 2000).

Traditional dietary fats, primarily composed of TAG, undergo digestion and metabolism to produce free fatty acids and monoglycerides, which are subsequently re‐esterified into TAG within the small intestine. These TAG molecules are then transported to the liver and adipose tissue as chylomicrons, which readily lead to energy surplus and fat accumulation (Kondo et al. 2003). However, as an essential nutrient, the complete restriction of dietary fat is neither practical nor scientifically sound. Therefore, the development of novel dietary lipids with metabolic regulatory functions has become a crucial strategy to address this dilemma. Among emerging alternatives, diacylglycerol (DAG) has garnered widespread attention due to its distinctive metabolic characteristics. Unlike TAG, the predominant isomer of DAG, 1,3‐DAG, undergoes digestion and yields significantly lower levels of re‐esterified TAG in intestinal epithelial cells, thereby reducing postprandial TAG levels (Meng et al. 2004). Clinical and animal studies have demonstrated that DAG can mitigate weight gain (Maki et al. 2002; Murase et al. 2001), reduce visceral fat deposition (Meng et al. 2004; Nagao et al. 2000), lower blood lipid levels (Ijiri et al. 2006; Yasukawa and Yasunaga 2001), regulate blood glucose (Eom et al. 2010; Zheng et al. 2015) and improve serum cholesterol (Ijiri et al. 2006; Yamamoto et al. 2006). Regarding its underlying mechanisms, DAG has been shown to influence β‐oxidation of fatty acids within the small intestine and liver or modulate the expression of hepatic lipogenesis‐related genes (Lu et al. 2020; Murase et al. 2005, 2002). However, its potential mechanisms at the hepatic lipid metabolite level remain unexplored. Investigating these metabolic alterations could unveil novel pathophysiological insights into obesity‐associated metabolic dysregulation, potentially informing targeted therapeutic interventions.

Lipids, as bioactive signaling molecules, play a vital role in metabolic homeostasis, and their dysregulated metabolic network is a hallmark of metabolic disorders such as obesity and non‐alcoholic fatty liver disease (Fahy et al. 2011; Perreault et al. 2016; Samuel and Shulman 2018). The liver, as the central regulatory organ of systemic lipid metabolism, maintains lipid homeostasis by integrating synthesis, degradation, and transport pathways. Hepatic lipidomics, through the systematic analysis of lipid species, abundance, metabolic flux, and dynamic equilibrium, not only elucidates the molecular nature of lipid metabolic disorders but also provides critical insights into the pathogenesis of metabolic diseases (Fabbrini and Magkos 2015; Ten Hove et al. 2020). Hepatic lipidomics offers a high‐resolution omics perspective for deciphering the metabolic regulatory mechanisms of dietary components. For instance, Feng et al. demonstrated through a comprehensive lipidomics study that the citrus flavonoid tangeretin significantly reduces hepatic lipid accumulation by modulating the metabolic network of fatty acids, diacylglycerols, triacylglycerols, ceramides, and cholesterol esters, thereby exerting lipid‐lowering and weight‐reducing effects (Feng et al. 2020). Similarly, phytosterols such as stigmasterol and β‐sitosterol have been shown to mitigate the progression of non‐alcoholic fatty liver disease by remodeling hepatic cholesterol metabolism, regulating the dynamic balance of polyunsaturated triacylglycerols, and altering the composition of free fatty acids (Feng et al. 2018). Thus, hepatic lipidomics, by precisely capturing dynamic lipid metabolic alterations, enables a systematic investigation of the molecular mechanisms underlying DAG's regulatory effects on hepatic lipid metabolism.

Corn oil, as a high‐quality edible oil, is highly digestible and possesses a clear appearance with a mild sensory profile, maintaining excellent physical stability under both cold and high‐temperature processing conditions. This makes it widely applicable in salad preparation and high‐temperature cooking (Dupont et al. 1990). Notably, compared to other vegetable oils, corn oil is not only rich in polyunsaturated fatty acids such as linoleic acid but also contains significantly higher levels of phytosterols than other common oils, granting it a distinct advantage in reducing atherosclerosis‐associated cholesterol levels (Lichtenstein et al. 1993; Maki et al. 2015; Ostlund et al. 2002; Wagner et al. 2001). Paradoxically, despite these benefits, corn oil demonstrates comparable limitations to saturated fat‐rich animal derivatives (e.g., lard) in adiposity regulation and weight management efficacy (Pavlisova et al. 2016). This selective metabolic regulation suggests that its primary mechanism may be concentrated on cholesterol metabolism pathways rather than exerting a comprehensive influence on energy balance. Therefore, developing functional oils with enhanced health benefits based on corn oil may further expand its advantages.

In this study, a combined clinical and animal experimental design was employed to systematically explore the lipid‐lowering and weight‐loss effects and mechanisms of action of corn‐based DAG oil. The clinical phase involved obese participants, during which the effects of DAG oil on anthropometric indicators and blood lipid profiles were comprehensively evaluated. In the animal phase, a high‐fat diet‐induced obese mouse model was established and integrated with hepatic lipidomic analysis to investigate the regulatory effects of DAG on hepatic lipid metabolite composition and abundance, as well as the expression of genes involved in lipid metabolism, thereby providing a theoretical foundation for the application of DAG as a functional dietary fat in lipid‐lowering and weight management strategies.

## 2 Materials and Methods

### 2.1 Clinical Trials

#### 2.1.1 Ethics

This study was conducted in accordance with the Declaration of Helsinki and was approved by the Ethics Committee of the Affiliated Hospital of Jiangnan University (Wuxi, China) under ethical number LS2023081. The study was conducted at the Affiliated Hospital of Jiangnan University (Wuxi, China) under the supervision of the attending physician, and all subjects were thoroughly briefed on the study and furnished written informed consent.

#### 2.1.2 Subject

Men and women with BMI ≥ 26.0 kg/m^2^ aged 18–65 years were recruited from Wuxi, China. These were included in the obese population with reference to the Chinese guidelines for the prevention and control of overweight and obesity among adults. Subjects were excluded if they regularly ingested antibiotics, corticosteroids, and vitamins. Subjects who lost or gained weight rapidly and were malnourished on daily intake were also excluded. Subjects with poor compliance and poor likelihood of follow‐up were also excluded from this study. A total of 94 subjects participated in this study.

#### 2.1.3 Test Oil

The experimental diacylglycerol oil (corn oil source) and the control corn oil, both in 500 mL/bottle, the oil and ingredients in the test product were food grade and supplied by Changshouhua Food Company Limited (Shandong, China). The composition of the test oils is shown in Table 1, where the control group was corn oil and the intervention group was DAG oil.

**TABLE 1:** Acylglycerol and fatty acid compositions of oil.

	Acylglycerol compositions [%]		
	Lard	Corn oil	DAG oil
TAG	95.71	94.48	35.87
1,2‐DAG	—	0.92	19.97
1,3‐DAG	1.16	1.57	43.87
Fatty acids (%)	3.13	3.03	0.29
C10:0	0.08	—	—
C11:0	0.02	—	—
C12:0	0.09	—	—
C13:0	0.01	—	—
C14:0	1.36	—	—
C15:0	0.04	—	—
C16:0	26.04	14.61	14.56
C16:1	2.07	0.12	0.10
C17:0	0.21	0.07	0.08
C17:1	0.17	0.04	0.03
C18:0	15.57	1.78	1.74
C18:1	40.64	28.02	28.16
C18:2	11.46	53.32	53.60
C20:0	0.08	0.11	0.40
C18:3	1.25	1.11	0.82
C20:1	0.27	0.53	0.26
C21:0	0.47	—	—
C22:0	0.08	—	—
C20:3	0.11	0.14	0.13
C24:0	—	0.15	0.12

#### 2.1.4 Design and Protocol

This study was a randomized, double‐blind, controlled clinical trial with a treatment period of 12 weeks. The primary outcomes were BMI, waist circumference, body fat percentage, serum total cholesterol (TC) levels, triglyceride (TG) levels, high‐density lipoprotein (HDL) level degrees, and low‐density lipoprotein (LDL) levels, and the secondary endpoints were indicators of hepatic and renal function and glycemic indicators. Subjects were asked to replace the frying oil with the provided product for cooking consumption, not exceeding 25 mL per day and recommended to be used 2 times a day. Subjects were measured once at weeks 0, 6, and 12 for observational indicators.

### 2.2 Animal Experiments

#### 2.2.1 Preparation of Feeding Oil

Corn‐based DAG oil and corn oil were both purchased from Changshouhua Food Company Limited (Shandong, China). The acylglycerol composition and main fatty acid profiles of DAG oil, corn oil, and lard are presented in Table 1, detailing the proportion of each fatty acid in the test oils. The results indicate that the fatty acid composition of DAG oil closely resembles that of corn oil. DAG oil consists of approximately 64% DAG and 36% TAG, with a 1,3‐DAG to 1,2‐DAG ratio of 44:20.

#### 2.2.2 Animal Experimental Design

Five‐week‐old male C57BL/6J mice were obtained from SPF (Suzhou) Biotechnology Co. Ltd. [Production License No. SCXK (Su) 2022‐0006]. All the animals were kept in a specific pathogen‐free setting at the Laboratory Animal Center of Jiangnan University (license no. SYXK (Su) 2016‐0045, Wuxi, China) under controlled conditions (temperature 23°C ± 2°C, relative humidity 50% ± 10%, 12/12‐h light–dark cycle). This experimental protocol was approved by the Laboratory Animal Ethics Committee of Jiangnan University (JN. No20230615c0321115 [277]). All animals underwent a 10‐day acclimatization period prior to experimental procedures, during which no manipulations were conducted.

C57BL/6J mice were randomly allocated into two groups: a control group (CON, *n* = 6) administered SYCON50H standard chow, and a modeling group (*n* = 18) fed a 45% high‐fat diet (SYHF45), both formulations procured from Jiangsu Synergy Pharmaceutical Biological Engineering Co. Ltd. All animals received ad libitum access to their respective diets during a 12‐week obesity induction phase. Following successful induction, obese mice underwent secondary randomization into three experimental groups (*n* = 6/group): (1) HFD group (continued SYHF45 diet), (2) CORN group (modified SYHF45 formulation with corn oil supplementation) and (3) DAG group (custom SYHF45 diet enriched with DAG oil). The CON group maintained baseline dietary conditions. All groups received their designated diets for a 10‐week intervention period with unrestricted water access. Tri‐daily measurements tracked feed consumption, water intake, and body mass. Table 1 details the nutritional composition of each experimental diet (Table 2).

**TABLE 2:** Composition of diets.

Ingredient	CON	High fat diets		
		HFD	CORN	DAG
**w/w (%)**				
DAG Oil	0.0	0.0	0.0	23.7
Corn Oil	2.4	2.9	23.7	0.0
Lard	1.9	20.7	0.0	0.0
Sucrose	16.8	20.7	20.7	20.7
Casein	19.0	23.4	23.4	23.4
Corn Starch	42.9	8.5	8.5	8.5
Cellulose	4.7	5.8	5.8	5.8
L‐Cystine	0.3	0.4	0.4	0.4
Maltodextrin	7.1	11.7	11.7	11.7
Mineral Mix	4.7	5.8	5.8	5.8
Vitamin	0.1	0.1	0.1	0.1
Energy, kcal/100 g	385.3	471.5	471.5	471.5
**Kcal (%)**				
Fat	10.3	45.1	45.1	45.1
Carbohydrate	69.9	35.1	35.1	35.1
Protein	19.8	19.8	19.8	19.8

*Note:* The energy value of each diet was calculated based on its macronutrient composition, with carbohydrate, protein, and fat contributing 4 kcal/g, 4 kcal/g, and 9 kcal/g, respectively.

At the end of the experiment, the mice were made to fast for 12 h, with water being provided ad libitum. Blood was collected from the orbital sinus and then executed, and the serum was analyzed biochemically immediately after it was obtained by centrifugation. All mice were dissected, and the liver, kidneys, abdominal, epididymal, and perirenal white adipose tissue were collected and weighed. A portion of the liver and abdominal white adipose tissue was fixed in 4% paraformaldehyde solution, while the remaining tissues were quickly frozen and then stored at −80°C.

#### 2.2.3 Serum Biochemical Analysis

Blood samples were centrifuged (3500 × g, 4°C, 15 min) and serum was isolated. Biochemical parameters including TG, TC, LDL, HDL, aspartate aminotransferase (AST), alanine aminotransferase (ALT), and superoxide dismutase (SOD) levels were quantified using a Mindray BS‐420 automated biochemical analyzer.

#### 2.2.4 Liver and White Adipose Tissue (WAT) Histological Analysis

For histomorphological evaluation, liver and adipose tissues fixed in 4% paraformaldehyde underwent sequential processing including dehydration, paraffin embedding, sectioning, and hematoxylin–eosin (H&E) staining. All stained tissue sections were subsequently subjected to comprehensive microscopic examination.

#### 2.2.5 Liver Lipid Extraction and Lipidomic Analysis

Hepatic tissue (~20 mg) was homogenized in 225 μL methanol via ice‐bath ultrasonication (1 min). The homogenate was combined with 750 μL methyl tert‐butyl ether, vortexed for 10 s, and incubated at ambient temperature for 30 min. Following the addition of 188 μL ultrapure water and vortexing (20 s), samples were incubated for 10 min and centrifuged (14,000 × g, 4°C, 15 min). Supernatants were evaporated under nitrogen at 30°C, then reconstituted in 100 μL isopropanol‐acetonitrile‐water (30:65:5, v/v/v) with vortexing (10 s). After final centrifugation (14,000 × g, 4°C, 10 min), clarified supernatants were retained for chromatographic analysis.

Hepatic lipidomics profiling was conducted via liquid chromatography‐electrospray ionization‐tandem mass spectrometry (LC‐ESI‐MS/MS). Chromatographic separation was achieved using a Kinetex XB‐C18 column (100 mm × 4.6 mm, 2.6 μm particle size) maintained at 30°C with a 0.3 mL/min flow rate. Mobile phase composition included: solvent A (methanol: acetonitrile: water, 1:1:3 v/v/v with 5 mM ammonium acetate) and solvent B (isopropanol with 5 mM ammonium acetate). The solvent gradient in volume ratios was as follows: 0–1 min, 30% B; 1–2 min, 30%–60% B; 2–5 min, 60% B; 5–10 min, 60%–70% B; 10–18 min, 70% B; 18–30 min, 70%–85% B; 30–35 min, 85%–98% B; 35–36 min, 98% B; 36–40 min, 30% B.

#### 2.2.6 RNA Isolation and qRT‐PCR Analysis

Liver tissue specimens were homogenized in 1 mL TRIzol reagent and transferred to nuclease‐free microcentrifuge tubes. Total RNA was isolated following manufacturer specifications, with concentration quantified and purity assessed via NanoDrop One (Thermo Fisher Scientific). Reverse transcription was performed using the HiScript III All‐in‐one RT SuperMix Kit (Vazyme, R333‐01). Quantitative PCR amplification employed ChamQ Universal SYBR qPCR Master Mix (Vazyme, Q711‐02) on a Bio‐Rad CFX384 platform. Relative mRNA expression levels were normalized to GAPDH reference values. Primer sequences utilized for qPCR amplification are detailed in Table S1.

### 2.3 Statistical Analysis

IBM SPSS Statistics and OriginPro 2022 were used for statistical analysis and data visualization, with results expressed as mean ± standard deviation (Mean ± SD). Clinical data were tested for normality, followed by independent samples t‐tests or Wilcoxon signed‐rank tests as appropriate. One‐way ANOVA with Duncan's post hoc test was applied for multiple group comparisons in animal experiments. SIMCA‐P 13.0 was used for principal component and partial least squares discriminant analyses of liver lipidomics. Differential lipids were identified based on VIP scores and *p*‐values, with KEGG enrichment conducted via MetaboAnalyst 5.0. Statistical significance was defined as *p* < 0.05.

## 3 Results

### 3.1 Clinical Trial

**FIGURE 1:**
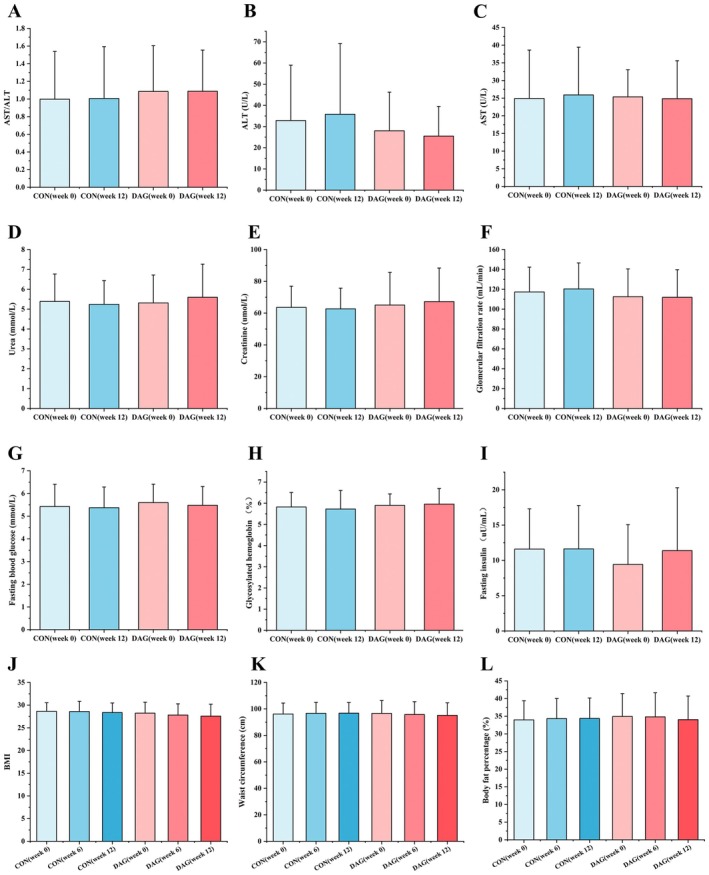
Indicators of liver and kidney function, glucose metabolism, and anthropometric indicators. Results are the mean ± SD (*N* = 47). Participants ingested either a TAG oil (CON group) or a DAG oil (DAG group). Unsigned annotation indicates no difference between groups.

As shown in Figure 1A–F, no statistically significant differences were observed in key liver function indicators (including AST, ALT, and AST/ALT ratio) or core renal function parameters (urea, creatinine, and glomerular filtration rate) between the DAG and CON groups before and after the intervention. This suggests that the product does not exert a significant impact on liver or kidney function during the intervention period, demonstrating a favorable safety profile. Further analysis of glucose metabolism‐related parameters (Figure 1G–I) revealed that fasting blood glucose, glycated hemoglobin, and fasting insulin levels remained stable (*p* > 0.05) in both groups before and after the intervention, indicating no significant regulatory effect of the product on glucose homeostasis. Regarding anthropometric measurements (Figure 1J–L), the BMI, waist circumference, and body fat percentage of participants in the DAG group exhibited no temporal variations before, during, or after the intervention (*p* > 0.05). Additionally, no significant differences were observed between the DAG and CON groups (*p* > 0.05), further confirming the absence of measurable effects on body composition.

**FIGURE 2:**
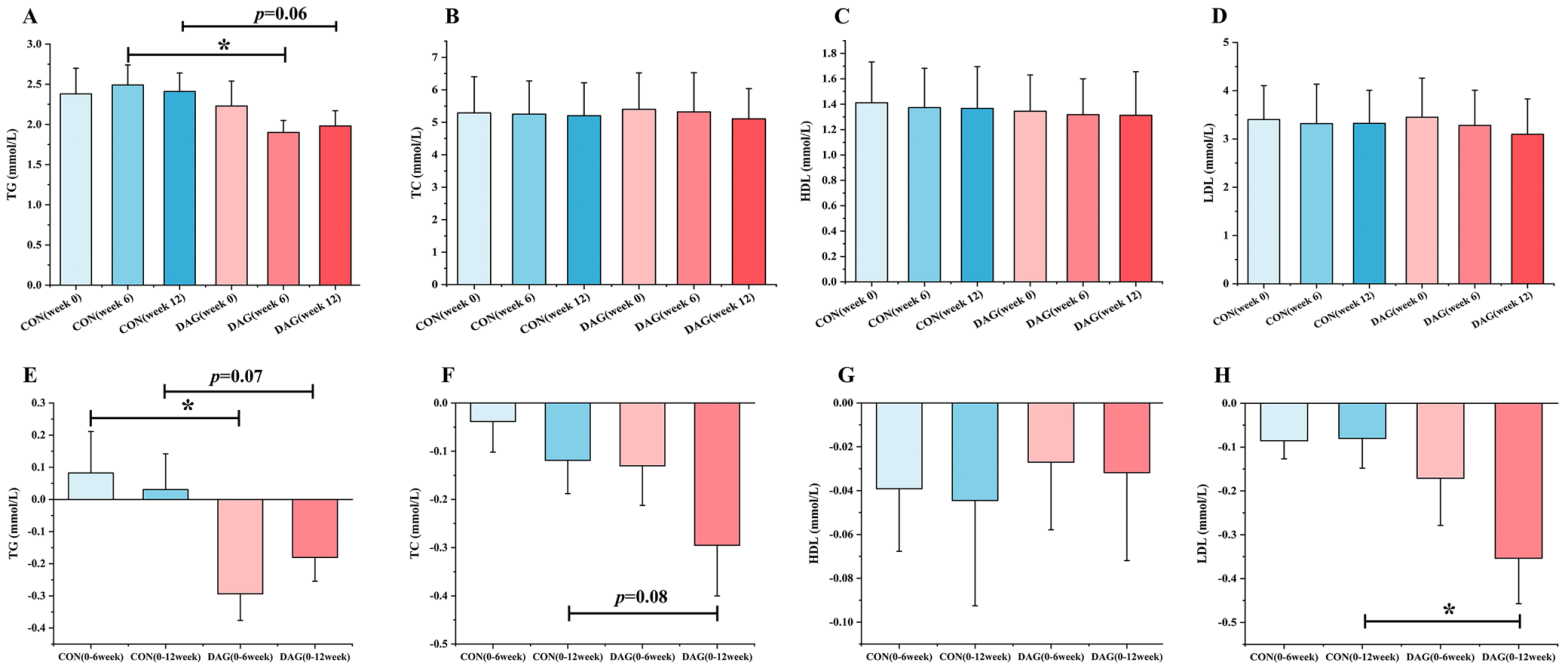
Clinical blood lipid levels. Values are the mean ± SD (*N* = 47). *denotes statistically significant differences (*p* < 0.05) determined by independent samples *t*‐test for inter‐group comparisons or Wilcoxon signed‐rank test for within‐group comparisons.

As shown in Figure 2A–D, there were no statistically significant differences between the DAG and CON groups in TC, HDL, or LDL levels before and after the intervention. However, a distinct improvement in TAG metabolism was noted: at week 6 of the intervention, the DAG group showed significantly lower TG levels than the CON group (*p* < 0.05), and this downward trend persisted until week 12 (DAG vs. CON, *p* = 0.06). Further time‐series analysis (Figure 2E–H) revealed that the TG reduction in the DAG group during weeks 0–12 was significantly greater than that in the CON group (*p* < 0.05), with a similar trend observed over the 0–12 week period (*p* = 0.07). Although the overall reduction in TC from week 0 to week 12 did not attain statistical significance (*p* = 0.08), a statistically significant between‐group difference was observed in LDL, with a greater reduction observed in the DAG group compared to the CON group (*p* < 0.05). The findings indicate that the lipid‐lowering effects of DAG intervention are indicator‐specific, primarily improving TAG and LDL dynamics, while its regulatory effects on TC and HDL are relatively limited.

### 3.2 Animal Experiments

#### 3.2.1 Body Weight, Food Intake and Adipose Tissue Weight

**FIGURE 3:**
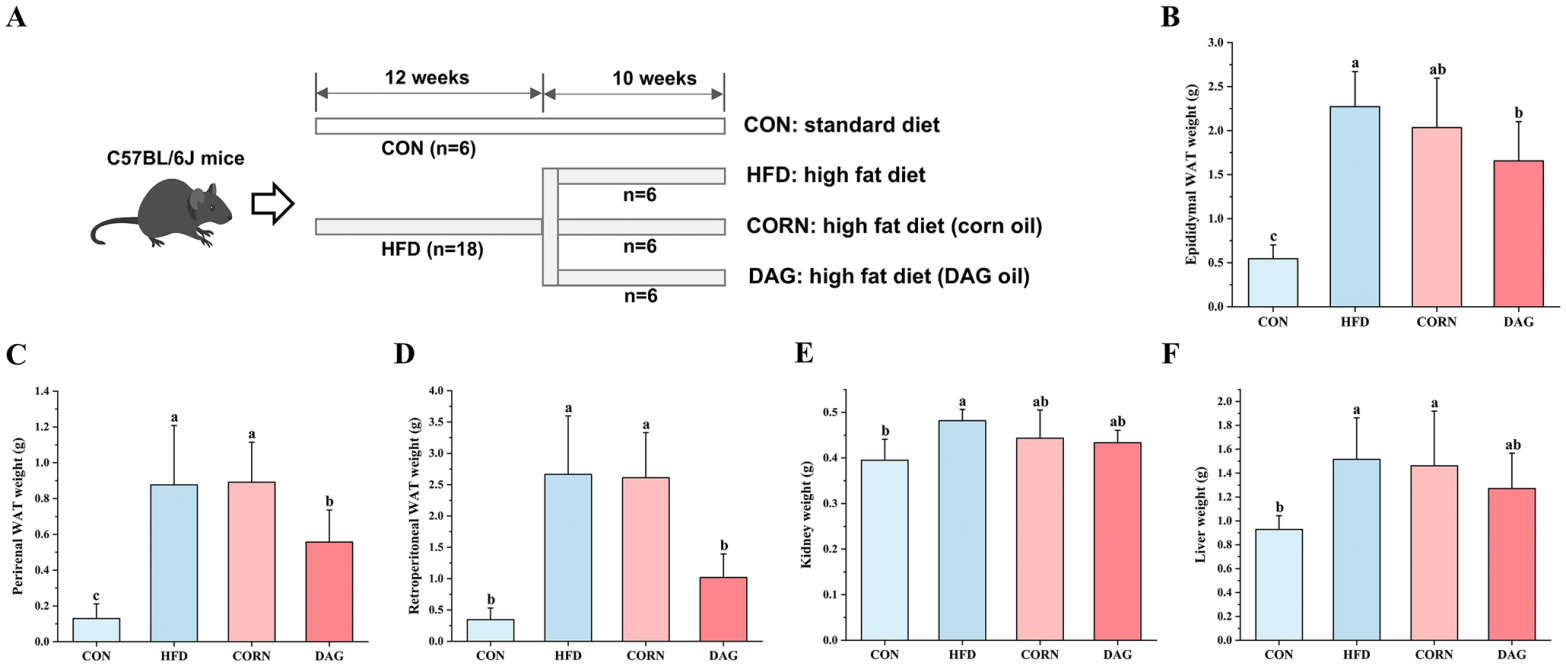
Schematic representation of obesity induction protocols and experimental grouping (A), with corresponding effects of DAG supplementation on weights of epididymal (B), perirenal (C), and retroperitoneal (D) white adipose tissue, along with hepatic (E) and renal organ weights in mice. Results are presented as mean ± SD (*n* = 6). Distinct alphabetical superscripts denote statistically significant differences determined by one‐way ANOVA (*p* < 0.05) with post hoc Duncan's multiple range test for intergroup comparisons.

This investigation evaluated the therapeutic efficacy of DAG supplementation in counteracting high‐fat diet‐induced obesity. As shown in Figure 3A, the experimental protocol comprised two sequential phases: First, a diet‐induced obesity murine model was established through 12‐week administration of lard‐based high‐fat chow (45% fat‐derived caloric contribution), with baseline body weights demonstrating no intergroup variance (*p* > 0.05). Subsequently, diet‐induced obese mice underwent stratified randomization into three experimental cohorts for a 10‐week dietary intervention protocol.

Experimental outcomes (Table 3) revealed that the DAG intervention group exhibited significantly attenuated terminal body weight gain compared to both HFD and CORN cohorts (*p* < 0.05), while no significant disparity emerged between HFD and CORN groups. Notably, comparable daily caloric intake across three high‐fat cohorts (*p* > 0.05) suggests DAG's weight‐modulatory mechanisms are dissociated from alterations in feeding behavior. Epididymal WAT mass demonstrated significant reduction in DAG‐treated mice relative to HFD controls (*p* < 0.05), though no significant variance versus CORN counterparts was observed (Figure 3B). Marked reductions in perirenal WAT and retroperitoneal WAT occurred in DAG‐supplemented animals compared to both HFD and CORN groups (*p* < 0.05) (Figure 3C,D). Hepatic and renal organ weights demonstrated no intergroup differences across experimental conditions (*p* > 0.05) (Figure 3E,F).

**TABLE 3:** Effects of different oils on body weight and food intake of mice.

	CON	HFD	CORN	DAG
Initial body weight (g)	22.78 ± 1.36	22.19 ± 0.92	22.16 ± 0.69	22.49 ± 1.01
Modeling weight (g)	30.18 ± 2.79^b^	37.06 ± 3.52^a^	36.67 ± 3.40^a^	36.58 ± 4.74^a^
Final body weight (g)	32.99 ± 2.59^c^	47.64 ± 1.03^a^	45.45 ± 5.44^a^	39.77 ± 3.23^b^
Daily feed consumption (g/per mouse/day)	3.36 ± 0.14^a^	2.77 ± 0.09^b^	2.80 ± 0.12^b^	2.84 ± 0.13^b^

*Note:* results are means ± SD (*n* = 6). *p* < 0.05 indicates a significant difference, which is indicated by different lowercase letters.

#### 3.2.2 Effect of DAG on Serum Biochemical Parameters in Mice

**FIGURE 4:**
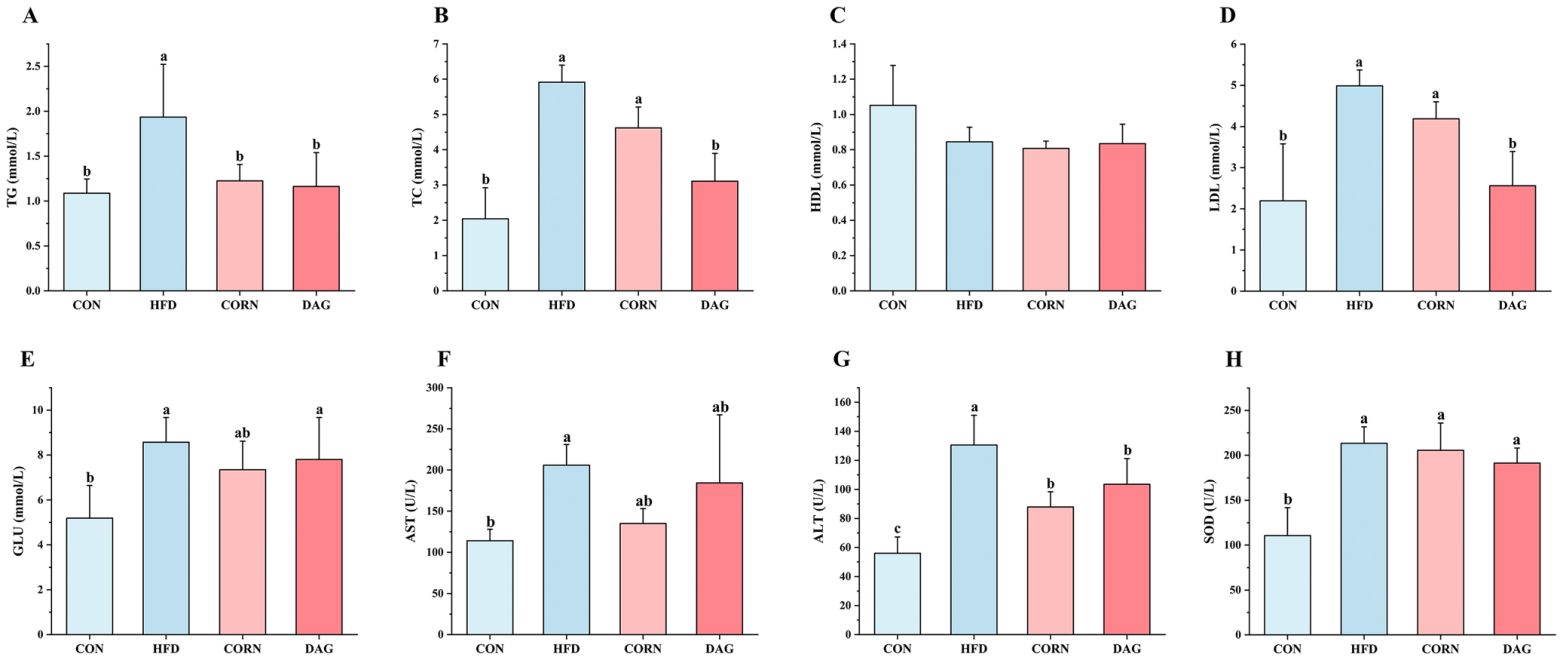
The impact of DAG supplementation on fasting serum biomarkers including TG (A), TC (B), HDL (C), LDL (D), GLU (E), AST (F), ALT (G) and SOD (H) levels. Data are presented as mean ± SD (*n* = 4). Distinct alphabetical superscripts denote statistically significant differences determined by one‐way ANOVA (*p* < 0.05) with post hoc Duncan's multiple range test for intergroup comparisons.

To elucidate DAG's regulatory effects on lipid metabolism, comprehensive serum metabolite profiling was conducted. As shown in Figure 4, compared with the HFD group, both CORN and DAG significantly reduced TG levels (*p* < 0.05), while no significant differences were observed among the CORN, DAG, and CON groups. DAG supplementation notably decreased TC and LDL concentrations versus both the HFD and CORN groups (*p* < 0.05), achieving levels comparable to the CON group (*p* > 0.05). HDL levels remained consistent across all groups (*p* > 0.05). While GLU levels showed no intergroup variation among high‐fat cohorts, CORN exhibited a marginal reduction approaching CON values. CORN and DAG slightly downregulated AST levels, with no significant difference compared to the HFD group and CON group. Both CORN and DAG significantly attenuated ALT levels relative to HFD (*p* < 0.05). High‐fat feeding universally elevated SOD activity (*p* < 0.05) without intergroup differences among fat‐modified diets.

#### 3.2.3 Effect of DAG on the Morphology of Liver and WAT

**FIGURE 5:**
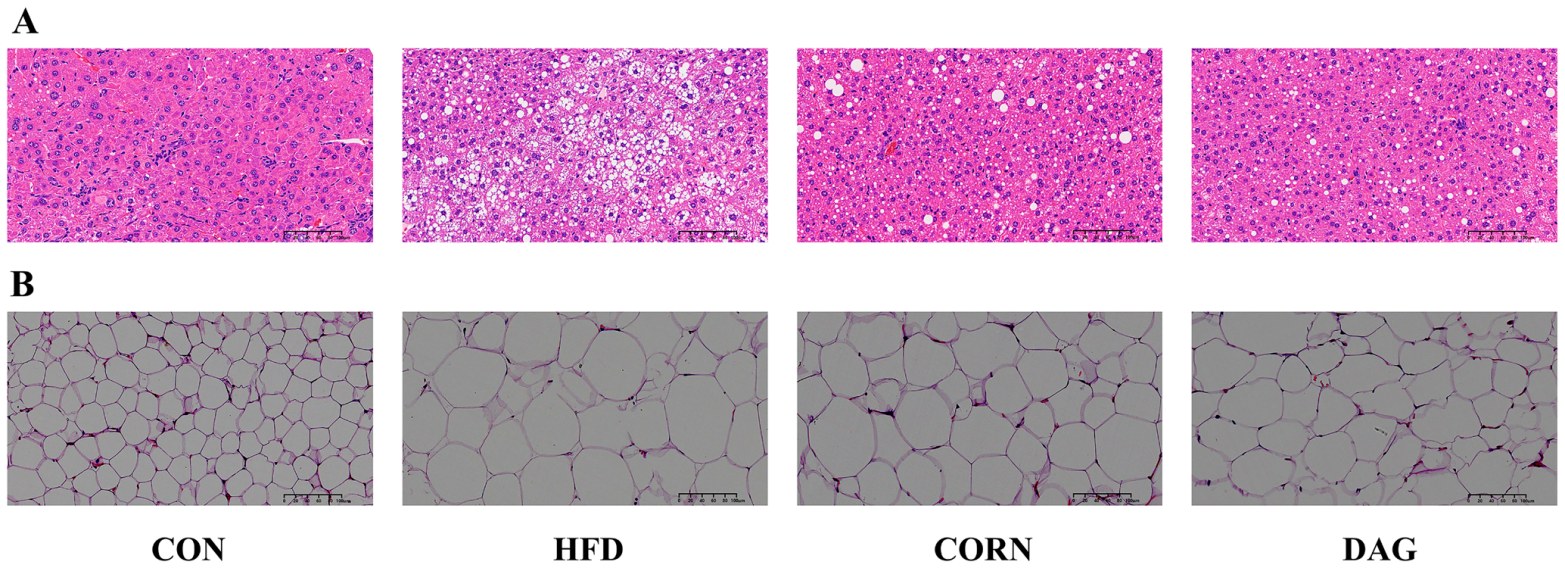
Representative H&E staining of the liver (A) and white adipose tissue (B) (200×). (*n* = 4).

To investigate the effects of DAG on liver steatosis and adipose deposition in obese mice, H&E staining was performed on liver and abdominal white adipose tissue sections to analyze their histological morphology. As shown in Figure 5A, in the CON group, hepatocytes were evenly arranged with normal eosinophilic cytoplasm, and no significant lipid vacuoles were observed. In the HFD group, hepatocyte density significantly increased, with multifocal lipid vacuoles present within the cytoplasm. The CORN group showed a decrease in hepatocyte density compared to the HFD group, with fewer lipid vacuoles, though regional distribution was still observed, suggesting partial reversal of steatosis. The DAG group significantly reduced vacuolar steatosis, with lipid vacuoles almost disappearing, and hepatocytes were arranged similarly to the CON group, demonstrating the most pronounced reversal of steatosis. Figure 5B shows that in the HFD group, adipocytes were significantly hypertrophied, with some areas exhibiting cell membrane rupture and lipid leakage. In the CORN group, adipocyte volume was markedly reduced compared to the HFD group, with partial restoration of cell membrane integrity. The DAG group exhibited adipocytes with volumes between those of the CORN and CON groups, with no lipid leakage, showing the best morphological improvement.

#### 3.2.4 Effects of DAG Supplementation on Hepatic Lipid Metabolism by Lipidomics Analysis

**FIGURE 6:**
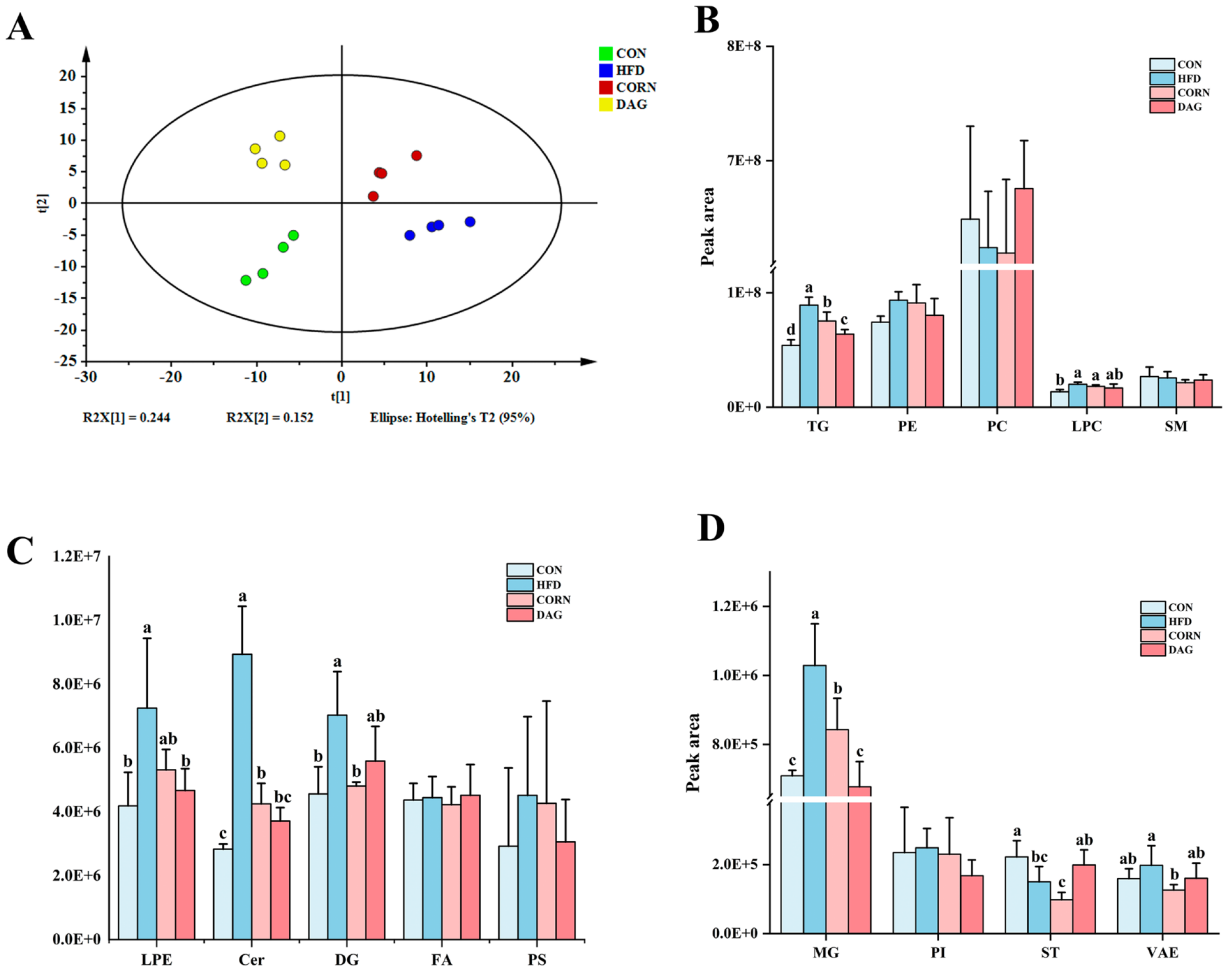
PCA score plot illustrating intergroup lipidomic divergence (A). Composition and quantitative profiles of identified hepatic lipid species (B‐D). Results are expressed as mean ± SD (*n* = 4). Distinct alphabetical superscripts denote statistically significant differences determined by one‐way ANOVA (*p* < 0.05) with post hoc Duncan's multiple range test for intergroup comparisons. Cer, Ceramides; DG, Diglyceride; FA, Fatty acyls; LPC, Lysophosphatidylcholine; LPE, Lysophosphatidylethanolamine; MG, Monoglyceride; PC, Phosphatidylcholine; PE, Phosphatidylethanolamine; PI, Phosphatidylinositol; PS, Phosphatidylserine; SM, Sphingomyelins; ST, Sterol lipids; TG, Triglyceride; VAE, Vitamin A fatty acid ester.

To further explore the potential mechanism of DAG in regulating lipid metabolism, lipidomics methods were used to analyze the metabolic changes of various lipids in the liver. Principal component analysis (PCA) is a commonly used unsupervised data analysis technique. Based on this, PCA analysis was performed on samples from four groups, in which liver lipids were determined by LC‐ESI‐MS/MS and the peak areas represented the contents. The results indicated that the samples of four groups exhibited obvious separation trends in the PCA plot (Figure 6A). There was a large degree of separation between the CON group and other groups. The distribution areas of the HFD group and the CORN group were relatively close, but there was still a certain degree of distinction. The DAG group was obviously separated from the other three groups, especially at a relatively far distance from the CON group and the HFD group, suggesting that DAG has significant differences in the composition of liver metabolites compared with other treatment groups, and may have a unique impact on liver lipid metabolism. Subsequently, supervised orthogonal partial least squares‐discriminant analysis (OPLS‐DA) was performed for each pair of groups. By integrating VIP values and S‐plot, the potential differences in metabolites between the two groups were elucidated (Figures S1–S6). The validation parameters (R^2^X, R^2^Y, and Q^2^) of the six OPLS‐DA models indicate good model fitness and predictive capability, enabling an effective assessment of lipidomic profile variations.

Quantitative analysis based on hepatic lipidomics identified 14 characteristic lipid molecules, with their total content distribution and intergroup variation patterns illustrated in Figure 6B–D. The HFD group led to significant lipid metabolic disorders. This was demonstrated by a statistically significant rise in the hepatic accumulation of six lipid classes, including TG, lysophosphatidylcholine (LPC), lysophosphatidylethanolamine (LPE), ceramide (Cer), diacylglycerol (DG), and monoacylglycerol (MG), when compared to the CON group (*p* < 0.05). Notably, the CORN group exhibited a certain degree of metabolic improvement, significantly reducing the abnormal accumulation of TG, Cer, DG, and MG (*p* < 0.05). The DAG oil intervention demonstrated even greater efficacy, not only effectively lowering TG, Cer, and MG levels compared to the HFD group (*p* < 0.05) but also significantly outperforming the corn oil group in TG and MG regulation (*p* < 0.05). Particularly noteworthy is the case of DG lipids—although DAG intervention did not achieve a statistically significant reduction in DG levels, no significant difference was observed between the DAG and CORN groups, suggesting that the metabolic regulatory effect of DAG oil closely parallels that of corn oil.

**FIGURE 7:**
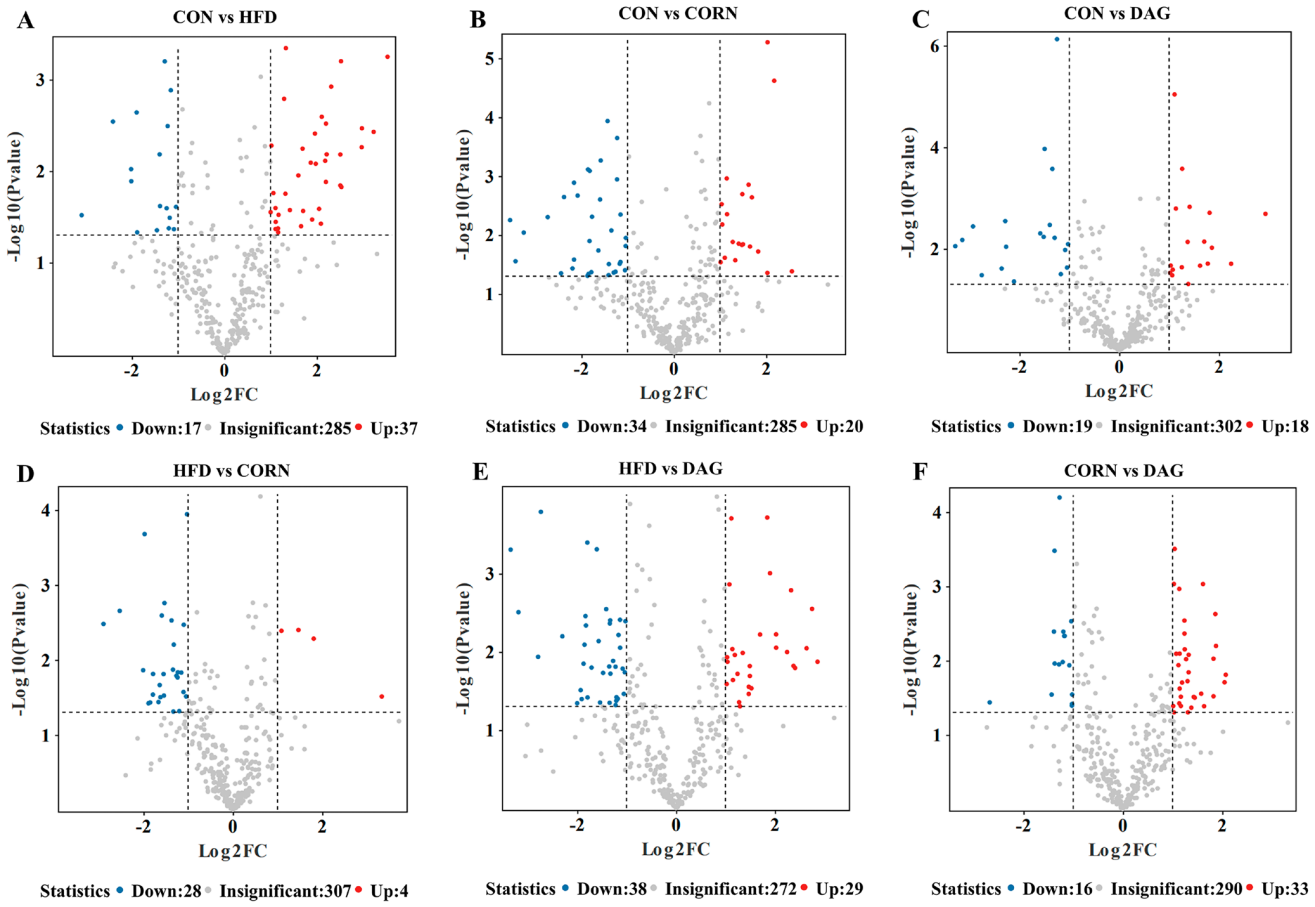
Volcanic diagram of differential metabolites between groups. (A‐F) Volcano plot of CON vs. HFD, CON vs. CORN, CON vs. DAG, HFD vs. CORN, HFD vs. DAG, and CORN vs. DAG, respectively. Up‐regulated and down‐regulated metabolites are indicated as red and blue dots, respectively. No significant changes are indicated as gray dots (*n* = 4).

According to the screening criteria of an absolute log_2_ fold change (FC) ≥ 1 and *p* < 0.05, the volcano plot analysis intuitively presents the quantitative variation characteristics of differential metabolites between groups (Figure 7). The dot distribution pattern in the volcano plot clearly illustrates the metabolite populations that meet the significance threshold, enabling the rapid identification of significantly upregulated or downregulated metabolites. Comparative analysis revealed that, compared with the CON group, the HFD group exhibited a significant downregulation of 17 lipids and an upregulation of 37 lipids. Meanwhile, the DAG intervention group showed an upregulation of 29 lipids and a downregulation of 38 lipids compared with the HFD group. Notably, compared with the CON group, the CORN group displayed a significant decrease in 34 lipids and an increase in 20 lipids, whereas the DAG group exhibited an upregulation of 33 lipids and a downregulation of 16 lipids compared with the CORN group. These findings suggest that DAG may reverse HFD‐ and CORN‐induced lipid metabolic disorders through multi‐target regulation.

**FIGURE 8:**
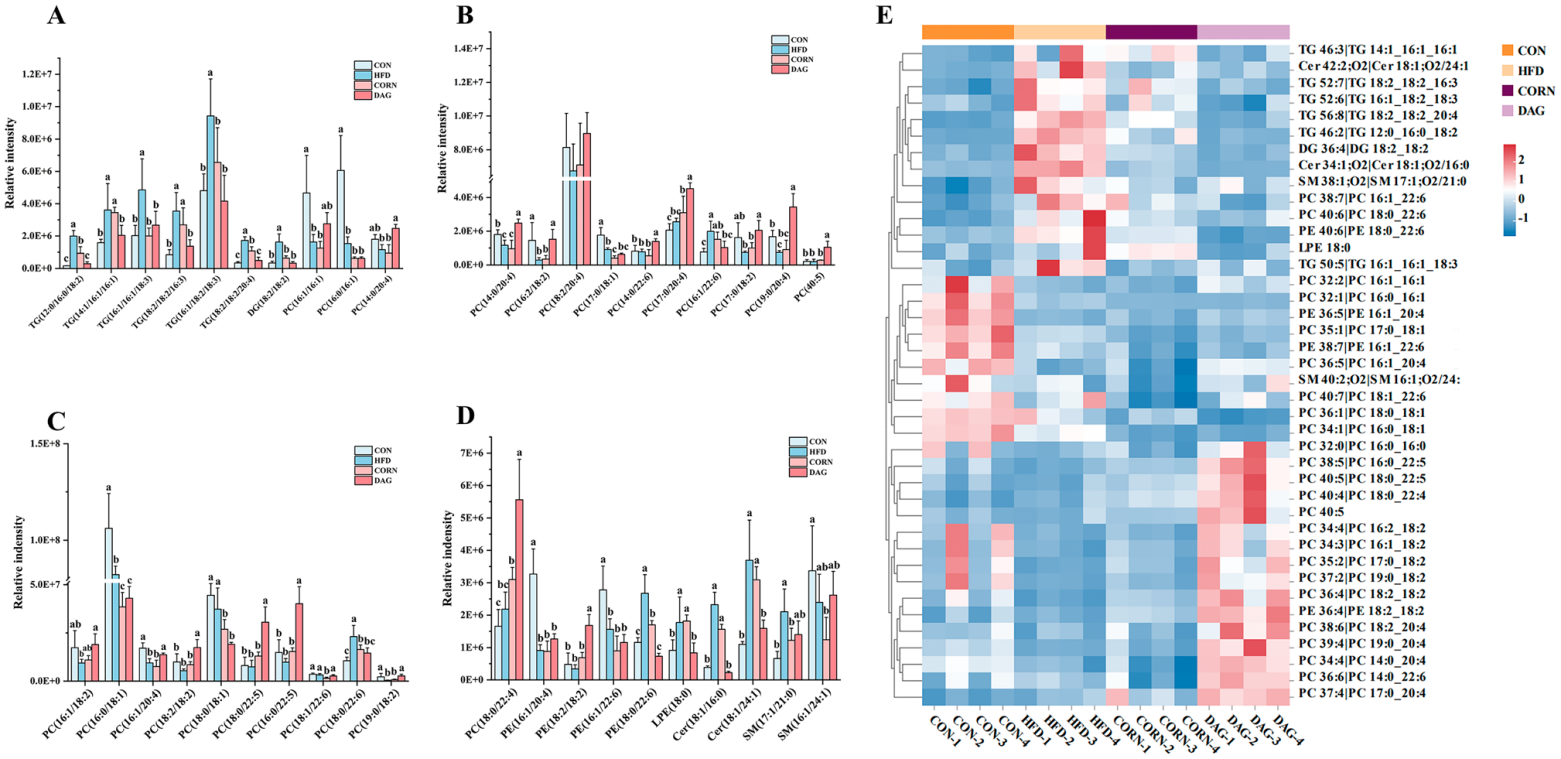
Bar charts (A–D) and heatmap (E) of differential metabolite contents. Results are expressed as mean ± SD (*n* = 4). Distinct alphabetical superscripts denote statistically significant differences determined by one‐way ANOVA (*p* < 0.05) with post hoc Duncan's multiple range test for intergroup comparisons. The colors represent the content of different lipid categories, with red indicating high lipid levels and blue indicating low levels.

Further screening based on the VIP value > 1 identified 40 key differential lipids. Hierarchical clustering analysis (Figure 8E) revealed their significant expression differences among the four groups. Specifically, the HFD group showed a significant increase in six TGs (TG (12:0/16:0/18:2), TG (14:1/16:1/16:1), TG (16:1/16:1/18:3), TG (18:2/18:2/16:3), TG (16:1/18:2/18:3) and TG (18:2/18:2/20:4)), DG (18:2/18:2), two PCs (PC (16:1/22:6) and PC (18:0/22:6)), PE (18:0/22:6), LPE (18:0), two Cers (Cer (18:1/16:0) and Cer (18:1/24:1)) and SM (17:1/21:0). The level of six PCs (PC (16:1/16:1), PC (14:0/20:4), PC (16:2/18:2), PC (19:0/20:4), PC (16:1/20:4) and PC (19:0/18:2)) was significantly reduced. DAG intervention repaired these lipid anomalies to levels equivalent to those in the CON group. In the CORN group, the contents of four TGs (TG (12:0/16:0/18:2), TG (14:1/16:1/16:1), TG (18:2/18:2/16:3) and TG (18:2/18:2/20:4)), PC (16:1/22:6), PE (18:0/22:6), LPE (18:0) and two Cers (Cer (18:1/16:0) and Cer (18:1/24:1)) were markedly enhanced in contrast to the CON group, while the contents of six PCs (PC (16:1/16:1), PC (14:0/20:4), PC (16:2/18:2), PC (16:1/20:4), PC (18:1/22:6) and PC (19:0/20:4)) significantly decreased. Similarly, DAG intervention bidirectionally regulated these lipid levels back to physiological ranges. Comprehensive analysis identified 6 TGs, 1 PE, 10 PCs and 1 LPE as significantly restored lipid species under DAG intervention, suggesting their potential role as biomarkers for DAG‐mediated hepatic lipid homeostasis regulation. These results offer essential perspectives on the molecular mechanisms through which DAG improves lipid metabolism.

#### 3.2.5 Pathway Analysis

**FIGURE 9:**
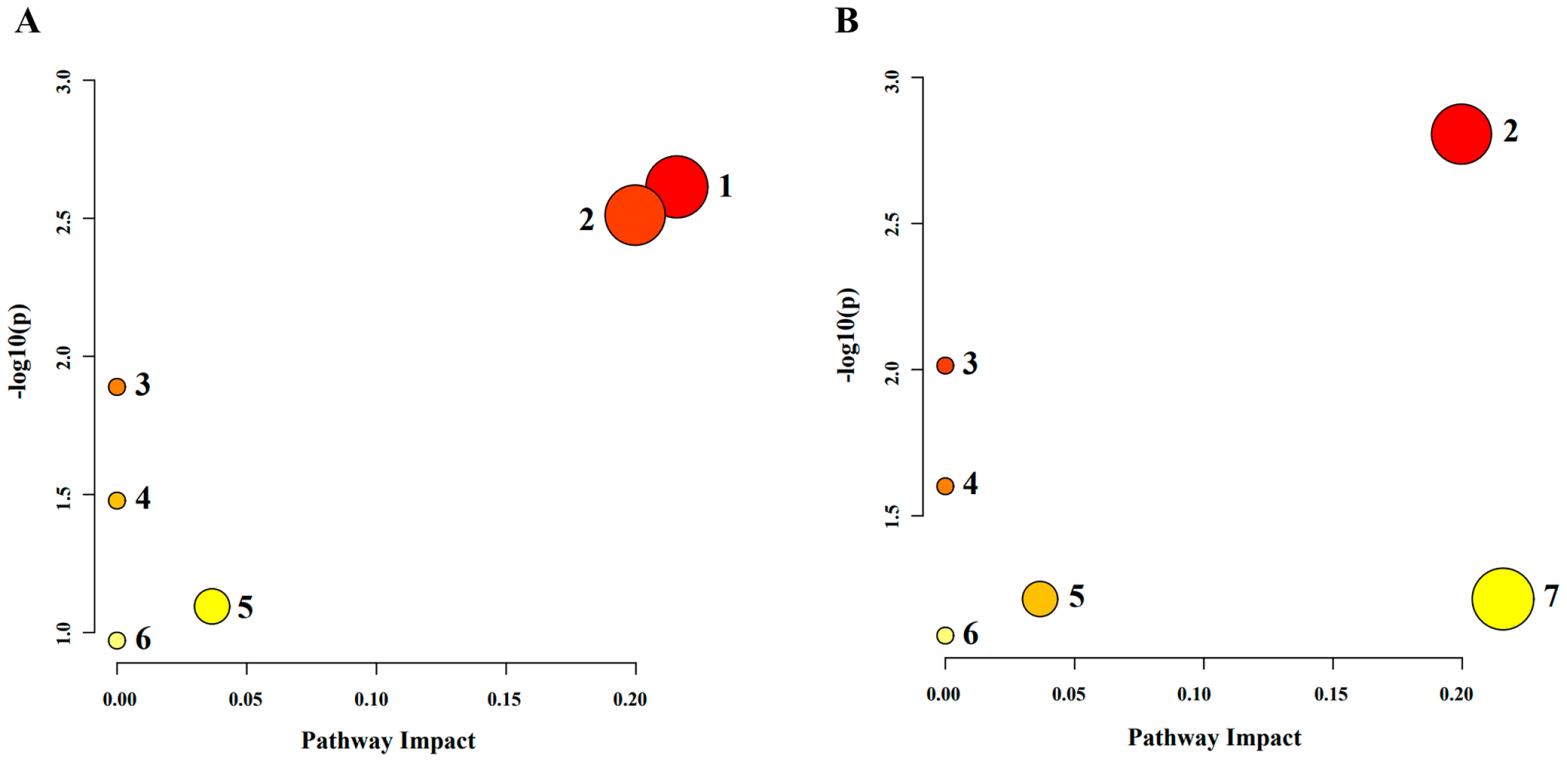
Lipid metabolism pathway analysis based on differential metabolites. (A) Comparison between the CON and HFD groups revealed alterations in key lipid metabolism pathways. (B) The metabolic pathways altered between the HFD vs. DAG and CORN vs. DAG groups were identical, indicating that both corn oil and DAG intervention influenced lipid metabolism through similar regulatory mechanisms. 1: Sphingolipid metabolism; 2: Glycerophospholipid metabolism; 3: Linoleic acid metabolism; 4: Alpha‐linolenic acid metabolism; 5: GPI‐anchor biosynthesis; 6: Arachidonic acid metabolism; 7: Sphingolipid metabolism.

Pathway enrichment analysis based on the identified differential lipid metabolites revealed potential metabolic pathways involved. As shown in Figure 9A, compared with the CON group, HFD‐fed mice exhibited alterations in the sphingolipid metabolic pathway, glycerophospholipid metabolism pathway, linoleic acid metabolic pathway, alpha‐linolenic acid metabolic pathway, glycosylphosphatidylinositol (GPI)‐anchor biosynthesis metabolic pathway, and arachidonic acid metabolic pathway, with the sphingolipid metabolic pathway and glycerophospholipid metabolism pathway being considered significantly affected based on *p* < 0.05 and Impact > 0. Notably, DAG intervention (Figure 9B) primarily exerted its regulatory effects on lipid metabolism through glycerophospholipid metabolism.

**FIGURE 10:**
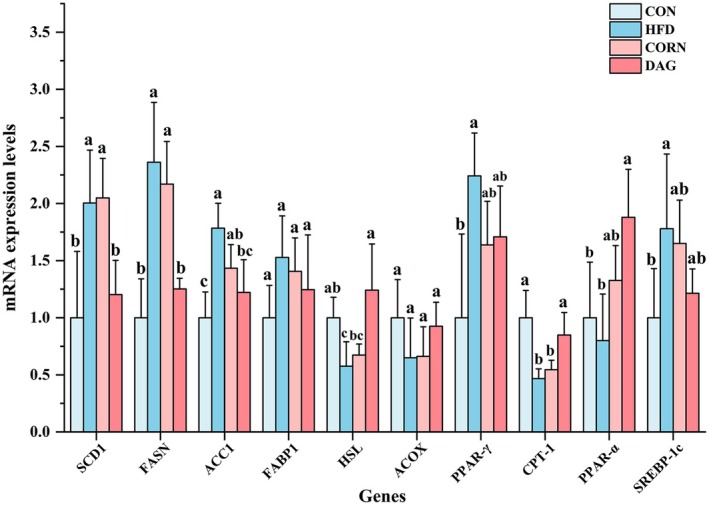
Hepatic lipid metabolism‐related gene expression levels. Data are presented as mean ± standard deviation (*n* = 4). Distinct alphabetical superscripts denote statistically significant differences determined by one‐way ANOVA (*p* < 0.05) with post hoc Duncan's multiple range test for intergroup comparisons. GAPDH expression was used as an internal control for normalization. ACC1, Acetyl‐CoA carboxylase 1; ACOX, Acyl‐CoA oxidase; CPT‐1, Carnitine palmitoyltransferase 1; FABP1, Fatty acid binding protein 1; FASN, Fatty acid synthase; HSL, Hormone‐sensitive lipase; PPAR‐α, Peroxisome proliferator‐activated receptor α; PPAR‐γ, Peroxisome proliferator‐activated receptor γ; SCD1, Stearoyl‐CoA desaturase 1; SREBP‐1c, Sterol regulatory element‐binding protein 1c.

#### 3.2.6 Effects of DAG Supplementation on Hepatic Lipid Metabolism by Lipidomics Analysis

To further investigate the potential mechanisms by which DAG regulates hepatic lipid metabolism, qRT‐PCR was used to quantify the expression levels of lipid metabolism‐related genes in the liver (Figure 10). Compared with the CON group, the HFD group exhibited significant changes in most genes except for fatty acid binding protein 1 (FABP1), acyl‐CoA oxidase (ACOX), and peroxisome proliferator‐activated receptor α (PPAR‐α), indicating lipid metabolic dysregulation. Compared with the HFD group, the DAG group significantly downregulated the expression of lipogenic genes, including stearoyl‐CoA desaturase 1 (SCD1), fatty acid synthase (FASN), and acetyl‐CoA carboxylase 1 (ACC1) (*p* < 0.05), while significantly upregulating the expression of lipolytic gene hormone‐sensitive lipase (HSL) and fatty acid oxidation‐related genes, including CPT‐1 and PPAR‐α (*p* < 0.05), thereby ameliorating lipid metabolic disorders. In contrast, the CORN group, compared with the CON group, significantly increased the expression of lipogenic genes (SCD1, FASN, and ACC1, *p* < 0.05) while markedly decreasing the expression of the fatty acid oxidation‐related gene CPT‐1 (*p* < 0.05). Notably, the DAG group, compared with the CORN group, exhibited a significant reduction in SCD1 and FASN expression and a marked elevation in CPT‐1 expression (*p* < 0.05), demonstrating the superior efficacy of DAG in improving lipid metabolism.

## 4 Discussion

Obesity, as a global health challenge, is closely associated with the onset and progression of various chronic metabolic diseases. In the exploration of dietary intervention strategies, oils rich in DAG have demonstrated unique metabolic regulatory potential (Rudkowska et al. 2005). In this study, a 12‐week DAG intervention significantly reduced fasting serum TAG and LDL concentrations in obese individuals, with a decreasing trend observed in TC levels, though BMI and body fat percentage showed no significant improvement. This result contrasts with previous long‐term intervention studies that reported significant reductions in body weight and visceral fat mass (Maki et al. 2002; Saito et al. 2016). A 24‐week intervention study conducted by Nagao et al. in overweight individuals employed a test oil containing 83% DAG, accounting for 20% of total dietary fat intake. The results demonstrated that reductions in body weight and fat mass were significantly greater in the DAG group compared to the TAG group. A slight decrease in serum TG levels was observed; however, the change did not reach statistical significance (Nagao et al. 2000). Hidetoshi and his colleagues conducted a 1‐year intervention trial of DAG oil (containing 85% DAG) in 312 overweight people and found that for those who consumed < 10% of total fat from test oils, BMI did not differ between the DAG and TAG groups (*p* > 0.05); for those who consumed 10% ~ 20% of total fat from test oils, compared to the TAG group, the BMI was significantly lower in the DAG group (*p* = 0.039); for those who consumed > 20% of total fat from test oils, the reduction in BMI was more pronounced (*p* = 0.032). Furthermore, their study after 1 year of DAG intervention similarly found no difference in waist circumference between groups. This suggests that DAG as a proportion of total fat intake can significantly influence the degree of BMI reduction (Kawashima et al. 2008). The DAG oil used in this study had a DAG content of 60%, but since the exact proportion of DAG oil consumed to total fat consumed per day by the subjects was missing in this study, we hypothesized that the lack of significant changes in BMI, waist circumference, and body fat percentage in this study was due to the fact that the proportion of DAG consumed per day to total fat in the test population did not reach the threshold for significant changes in body composition. The improvement of body weight and body fat by DAG may be time‐dependent, and its physiological effects through regulating the dynamic balance of lipid metabolism may need to be accumulated over a longer period of time.

In the animal experiment analysis, compared to the HFD group (lard‐based) and the CORN group (corn oil‐based), the DAG group exhibited significantly lower fasting serum TG, TC, and LDL levels (*p* < 0.05), aligning with previous findings on DAG's regulatory effects on lipid metabolism (Anikisetty et al. 2018; Hue et al. 2009). Regarding liver function indicators, serum ALT levels in both the CORN and DAG groups were significantly lower than those in the HFD group (*p* < 0.01), though no statistical difference was observed between the two groups (*p* > 0.05). In white adipose tissue analysis, the epididymal and retroperitoneal fat weights in the DAG group were significantly lower than those in the CORN group (*p* < 0.05). Combined with the H&E staining results showing a reduction in adipocyte size, this suggests that DAG may inhibit excessive fat accumulation by modulating lipid droplet homeostasis, contrasting with the classical theory that WAT stores TAG through hypertrophy under energy surplus conditions (Wronska and Kmiec 2012). Liver pathology analysis further demonstrated that the DAG group exhibited attenuation of steatosis and preservation of tissue structure compared to the CORN group, confirming its specific regulatory effect on hepatic lipid metabolism disorders.

This study systematically analyzed hepatic lipid composition using lipidomics technology, investigating the characteristics of liver lipid metabolism in diet‐induced obese mice and the regulatory effects of DAG intervention. The results indicated a significant increase in the total levels of TG, LPC, LPE, Cer, DG, and MG in the HFD group, suggesting that high‐fat diet‐induced obesity leads to abnormal hepatic lipid accumulation. Both corn oil and DAG oil interventions showed a certain degree of improvement in hepatic lipid disorders. Notably, the reductions in TG and MG levels were more pronounced in the DAG group than in the CORN group, highlighting the superior efficacy of DAG intervention. Further analysis of key differential lipid metabolites revealed distinct intergroup variations. In the CORN group, four TGs, two Cers, one PE, and one LPE exhibited significantly higher levels than in the CON group, whereas six PCs showed a significant decrease. These lipids demonstrated a marked reversal in the DAG group, suggesting a regulatory effect of DAG on hepatic lipid composition. While excessive hepatic TAG accumulation is often associated with nonalcoholic fatty liver disease, emerging perspectives suggest that increased TAG storage may instead offer protection against FA‐mediated hepatotoxicity. The exact relationship between TAG accumulation and hepatocellular injury remains to be fully elucidated (Alves‐Bezerra and Cohen 2017; Semova and Biddinger 2021). However, under normal physiological conditions, the liver stores only a small amount of TAG, maintaining a homeostatic TAG content below 5% (Browning et al. 2004). Therefore, from the perspective of metabolic homeostasis under normal conditions, a significant increase in hepatic TG content at least indicates an abnormality in liver lipid metabolism. Cer exerts its biological effects by regulating cell proliferation, differentiation, and apoptosis, interacting with various pathways related to insulin resistance, oxidative stress, inflammation, and cell death. An increase in PE may promote lipid droplet aggregation and enlargement, potentially leading to hepatic steatosis (Mashek 2021). LPE may contribute to lipid droplet formation by inhibiting lipolysis and fatty acid biosynthesis, thereby playing a pathological role in the development of fatty liver (Yamamoto et al. 2022). Moreover, PC, as a major component of eukaryotic cell membranes and a key element in hepatic lipoprotein secretion, holds significant physiological importance (Skipski et al. 1967; Van Meer et al. 2008). Impaired PC biosynthesis diminishes hepatic very‐low‐density lipoprotein secretory capacity, thereby disrupting TAG efflux mechanisms and promoting pathological intrahepatic TAG accumulation (Cole et al. 2012). Consequently, DAG intervention significantly ameliorated hepatic lipid composition in high‐fat diet‐induced obese mice, effectively attenuating the severity of steatosis through targeted metabolic modulation.

The differences in liver lipid composition among the four groups of mice are a result of lipid metabolism regulation. QRT‐PCR technology has analyzed the regulatory pathways of key genes involved in liver lipid metabolism. In terms of the lipid synthesis pathway, the expression of the PPAR‐γ gene in the HFD group was significantly upregulated compared to the CON group (*p* < 0.05). This transcription factor can promote TAG synthesis by activating the SREBP‐1c/FASN signaling axis (Chen et al. 2023; Zhu et al. 1993). Although PPAR‐γ levels tended to decrease in both the CORN and DAG groups compared to the HFD group (*p* > 0.05), only the DAG intervention restored them to levels that were not statistically different from those of the CON group. SREBP‐1c is a transcription factor for adipose adipogenesis in the liver and is involved in regulating the expression of ACC1 and FASN genes (Dentin et al. 2005; Ferre and Foufelle 2007, 2010). SREBP‐1c expression is transcriptionally controlled by various nutritional and hormonal factors, with insulin being one of the most potent activators (Foretz et al. 1999; Shimomura, Bashmakov, Ikemoto, et al. 1999). SREBP‐1c levels are usually over‐induced in the liver of obese animals (Kammoun et al. 2009; Shimomura, Bashmakov, and Horton 1999; Shimomura et al. 2000). This same result was obtained in the present study, where SREBP‐1c levels were significantly higher in the HFD group than in the CON group. Elevated levels of hepatic SREBP‐1c lead to an elevated rate of hepatic fatty acid synthesis, which in turn leads to steatosis in mice (Shimomura, Bashmakov, and Horton 1999). ACC1 is one of the key enzymes in the hepatic de novo synthesis of fatty acids, and the malonyl coenzyme A catalytically generated by it is one of the substrates for the subsequent fatty acid synthesis catalyzed by FASN (Mao et al. 2006). FASN catalyzes the final step in fatty acid biosynthesis and is considered to be a major determinant of the liver's maximum capacity to produce FA through ab initio lipogenesis (Chirala and Wakil 2004; Menendez and Lupu 2007). The expression of FASN correlates significantly with the degree of hepatic steatosis (Dorn et al. 2010). Compared with the other two high‐fat groups, the DAG intervention significantly reduced the expression levels of SCD1 and FASN, and although the reductions in the levels of SREBP‐1c and ACC1 did not reach significance, they also decreased to levels similar to those of the CON group. FABP1 overexpression enhances fatty acid uptake, whereas ablation of the FABP1 gene reduces hepatic fatty acid uptake (Martin et al. 2003; Newberry et al. 2003). In the present study, there was no significant difference in FABP‐1 gene expression levels among the four groups. In terms of lipolysis, activation of PPAR‐α upregulates genes involved in peroxisomal and mitochondrial fatty acid β‐oxidation (including CPT‐1 and ACOX) to promote fatty acid uptake, utilization, and catabolism (Mandard et al. 2004; Rakhshandehroo et al. 2010). In this study, there was no significant difference in PPAR‐α levels between the CON group and the HFD and CORN groups, but the DAG group significantly increased PPAR‐α levels compared with the CON and HFD groups, suggesting that DAG can additionally increase the expression of PPAR‐α to a certain extent, which affects the expression of its downstream genes. As a nuclear receptor, PPAR‐α is activated by ligands such as long‐chain fatty acids, forms a heterodimer with retinoid X receptor, binds to the PPAR response element in the promoter region of the CPT‐1 gene, directly upregulates the mRNA expression of hepatic CPT‐1, enhances the transport capacity of fatty acids to mitochondria, and initiates β‐oxidation (Schlaepfer and Joshi 2020). The level of CPT‐1 was significantly reduced in the HFD and CORN groups, which suggests that the fatty acid oxidation capacity of the liver was impaired in obese mice under a high‐fat diet. DAG intervention significantly upregulated CPT‐1 expression, thereby enhancing hepatic fatty acid oxidation under a high‐fat diet and reducing hepatic lipid accumulation. The level of ACOX did not show any significant difference among the four groups for the time being. HSL mediated the cytoplasmic hydrolysis of TAG (lipolysis) and cholesteryl esters (Kraemer and Shen 2002). Compared to the other two high‐fat groups, DAG intervention significantly increased HSL expression levels. Thus, the systematic alterations in gene expression profiles described above confirm that DAG plays a central role in hepatic lipid metabolism through dual regulation: on the one hand, it significantly down‐regulates the gene expression of fatty acid synthesis‐related enzymes (e.g., FASN, SCD1); and on the other hand, it continuously activates the transcriptional activity of key enzymes for fatty acid β‐oxidation (CPT‐1). This multi‐target regulatory network effectively alleviated hepatic lipotoxicity, providing key evidence at the transcriptional level that DAG ameliorates systemic metabolic disorders.

## 5 Conclusion

In this study, we systematically analyzed the molecular mechanism of DAG as a dietary intervention to regulate lipid metabolism disorders, and verified its lipid‐lowering and weight‐loss efficacy with the help of population and animal experiments. In the clinical study, 12 weeks of DAG intervention significantly optimized the lipid status of obese people, with significant reductions in serum triglyceride, low‐density lipoprotein cholesterol and total cholesterol levels. In a mouse model of diet‐induced obesity, long‐term DAG intervention demonstrated multiple metabolic improvements: significant inhibition of body weight gain rate, reduction of fasting lipid levels, and reduction of white fat accumulation. Hepatic lipidomics showed that DAG normalized hepatic lipid composition in mice fed a high‐fat diet, with significant reductions in triglycerides, ceramides and monoacylglycerol, and the sterolipid content was brought back to the normal range. Pathway analyses based on differential lipids showed that DAG affected hepatic lipid composition mainly by intervening in the glycerophospholipid metabolism pathway. In addition, DAG intervention significantly inhibited gene expression of stearoyl‐CoA desaturase 1 and fatty acid synthase, while significantly upregulating gene expression of carnitine palmitoyltransferase 1. Thus, DAG can effectively improve liver lipid metabolism induced by high‐fat diet through the dual regulation mode of “inhibiting synthesis and promoting catabolism and oxidation”. In the future, it is necessary to further analyze the specific role of DAG isomers and verify their long‐term metabolic benefits by extending the intervention period and increasing the proportion of DAG intake, so as to promote the translational application of functional lipids in the prevention and treatment of chronic metabolic diseases.

## Author Contributions

**Lina Shi:** data curation (equal), investigation (lead), methodology (lead), software (equal), writing – original draft (equal). **Yiran Liu:** data curation (equal), software (equal), writing – original draft (equal). **Yuanyuan Yan:** conceptualization (lead), resources (equal). **Dongsheng Bian:** formal analysis (lead), project administration (equal). **Jun Jin:** supervision (equal), writing – review and editing (lead). **Qingzhe Jin:** project administration (equal), supervision (equal), writing – review and editing (supporting). **Jiai Yan:** project administration (equal), resources (equal), supervision (equal). **Xingguo Wang:** funding acquisition (lead), project administration (equal), supervision (equal).

## Ethics Statement

Clinical study was conducted in accordance with the Declaration of Helsinki and was approved by the Ethics Committee of the Affiliated Hospital of Jiangnan University (Wuxi, China) under ethical number LS2023081, dated 19 December 2023. All subjects were thoroughly briefed on the study and furnished written informed consent. The animal experiments and all procedures were approved by the Laboratory Animal Ethics Committee of Jiangnan University, under approval number JN.No20230615c0321115[277], dated 26 June 2023.

## Consent

The study was conducted in accordance with the Declaration of Helsinki and approved by the Ethics Committee of the Affiliated Hospital of Jiangnan University (Approval number: LS2023081, issued on 19 December 2023).

## Conflicts of Interest

The authors declare no conflicts of interest.

## Supporting information


**Table S1.** The primer sequences in the research.
**Figure S1.** The OPLS ‐ DA score plot and the loading plot based on VIP index between CON group and HFD group.
**Figure S2.** The OPLS ‐ DA score plot and the loading plot based on VIP index between CON group and CORN group.
**Figure S3.** The OPLS ‐ DA score plot and the loading plot based on VIP index between CON group and DAG group.
**Figure S4.** The OPLS ‐ DA score plot and the loading plot based on VIP index between HFD group and CORN group.
**Figure S5.** The OPLS ‐ DA score plot and the loading plot based on VIP index between HFD group and DAG group.
**Figure S6.** The OPLS ‐ DA score plot and the loading plot based on VIP index between CORN group and DAG group.

## Data Availability

The original contributions presented in the study are included in the article; further inquiries can be directed to the corresponding authors.
