# Supplementary material for: Hepatic Lipidomics Unravels the Lipid‐Lowering and Anti‐Obesity Efficacy of Diacylglycerol Oil: Mechanistic Insights From High‐Fat Diet‐Induced Obese Mice
Source: Food Sci Nutr. 2025 Jun 13;13(6):e70395. doi: 10.1002/fsn3.70395 (PMC12165833; doi:10.1002/fsn3.70395)
Supplement: Supplementary file 1 — Table S1. The primer sequences in the research. Figure S1. The OPLS ‐ DA score plot and the loading plot based on VIP index between CON group and HFD group. Figure S2. The OPLS ‐ DA score plot and the loading plot based on VIP index between CON group and CORN group. Figure S3. The OPLS ‐ DA score plot and the loading plot based on VIP index between CON group and DAG group. Figure S4. The OPLS ‐ DA score plot and the loading plot based on VIP index between HFD group and CORN group. Figure S5. The OPLS ‐ DA score plot and the loading plot based on VIP index between HFD group and DAG group. Figure S6. The OPLS ‐ DA score plot and the loading plot based on VIP index between CORN group and DAG group. [file FSN3-13-e70395-s001.docx]

**Table S1. The primer sequences in the research**

| Primer | Sequence (5’-3’) |
| --- | --- |
| PPAR-α fwd | GGATGTCACACAATGCAATTCGCT |
| PPAR-α rev | TCACAGAACGGCTTCCTCAGGTT |
| PPAR-γ fwd | TGTGGACCTCTCCGTGATGG |
| PPAR-γ rev | GGTTCTACTTTGATCGCACTTTGG |
| FASN fwd | CCGTCGTCTATACCACTGCT |
| FASN rev | GGCAAAGCTGGTGTCATCAA |
| ACC1 fwd | TGAATCTCACGCGCCTACTATG |
| ACC1 rev | ATGACCCTGTTGCCTCCAAAC |
| FABP1 fwd | CAGAGCCAGGAGAACTTTGAG |
| FABP1 rev | GATTTCTGACACCCCCTTGATG |
| SREBP-1c fwd | GGCACTAAGTGCCCTCAACCT |
| SREBP-1c rev | GCCACATAGATCTCTGCCAGTGT |
| SCD1 fwd | CCAGTTCTTACACGACCACCAC |
| SCD1 rev | CCCGAAGAGGCAGGTGTAGA |
| HSL fwd | CTCACAGTTACCATCTCACCTC |
| HSL rev | GATTTTGCCAGGCTGTTGAGTA |
| ACOX fwd | AGGGAATTTGGCATCGCAG |
| ACOX rev | GATTCAGCAAGGTAGGGATAAACA |
| CPT-1 fwd | GCACGAGGAAAAAATAAGCAATCT |
| CPT-1 rev | TTGTCAAACCACCTGTCGAAAC |
| GAPDH fwd | CAACTCCCACTCTTCCACCT |
| GAPDH rev | GAGTTGGGATAGGGCCTCTC |


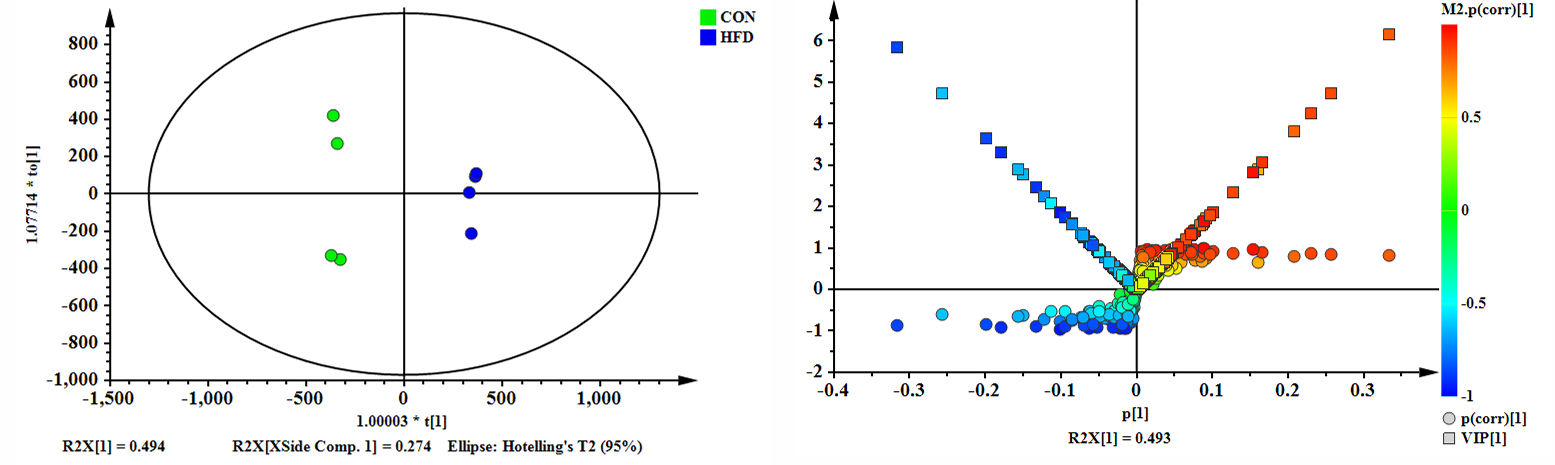


Figure S1. The OPLS - DA score plot and the loading plot based on VIP index between CON group and HFD group


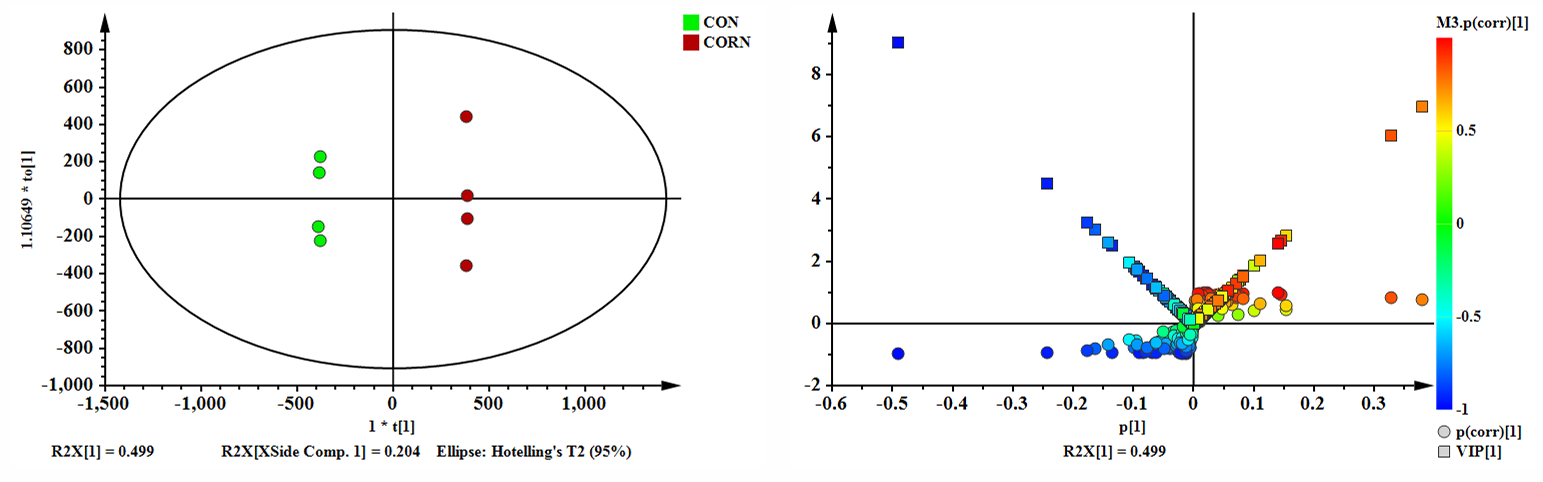


Figure S2. The OPLS - DA score plot and the loading plot based on VIP index between CON group and CORN group


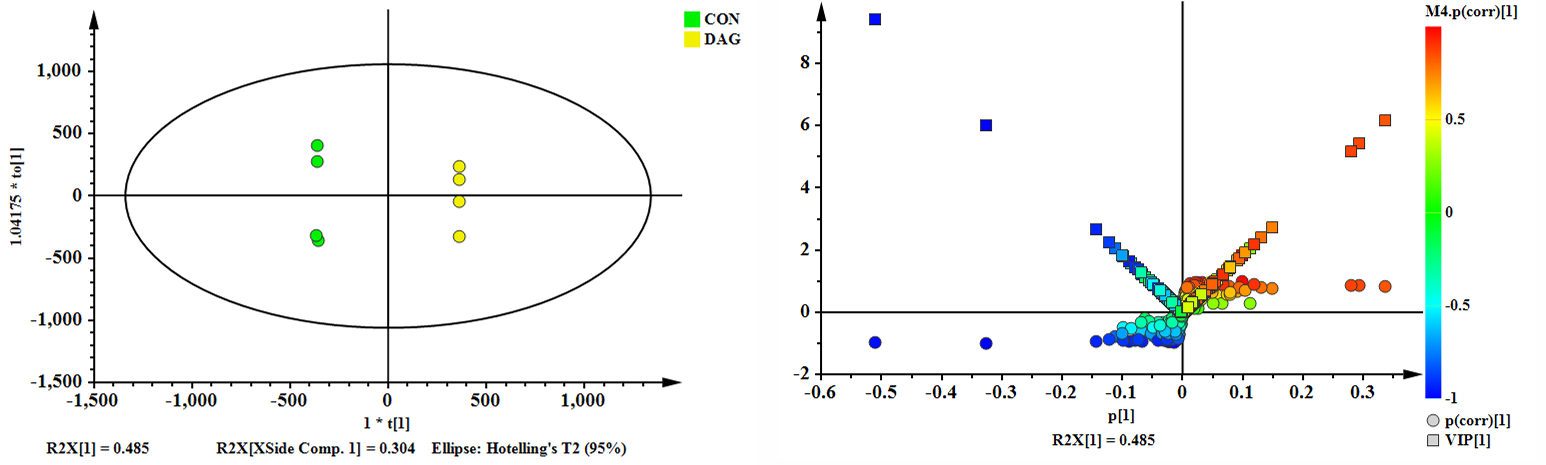


Figure S3. The OPLS - DA score plot and the loading plot based on VIP index between CON group and DAG group


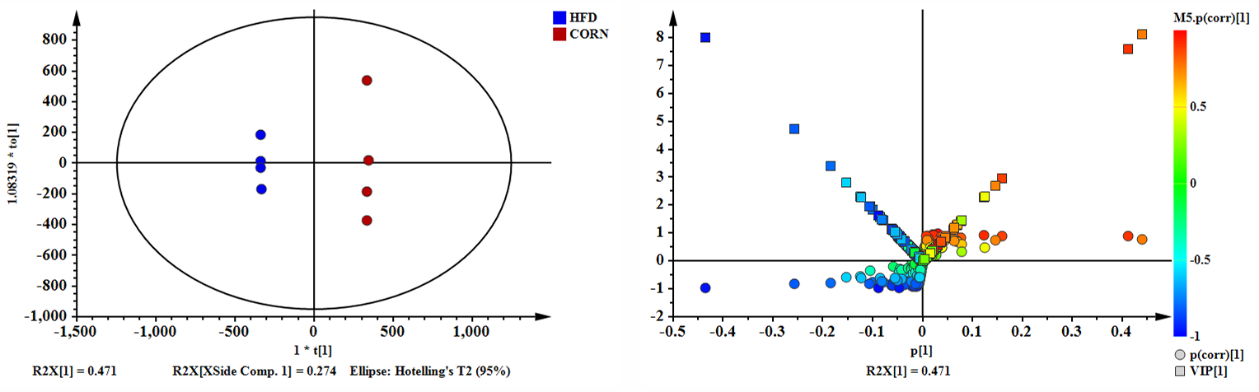


Figure S4. The OPLS - DA score plot and the loading plot based on VIP index between HFD group and CORN group


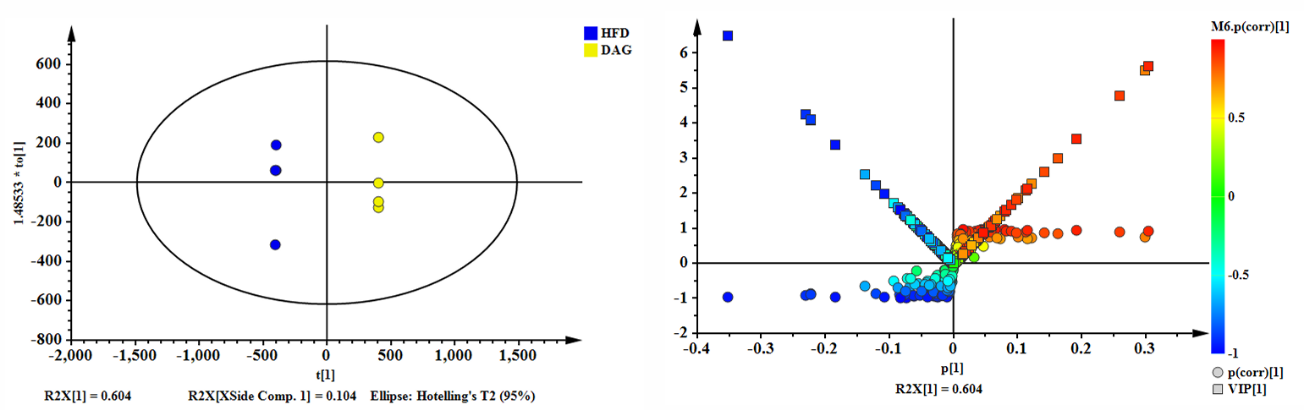


Figure S5. The OPLS - DA score plot and the loading plot based on VIP index between HFD group and DAG group


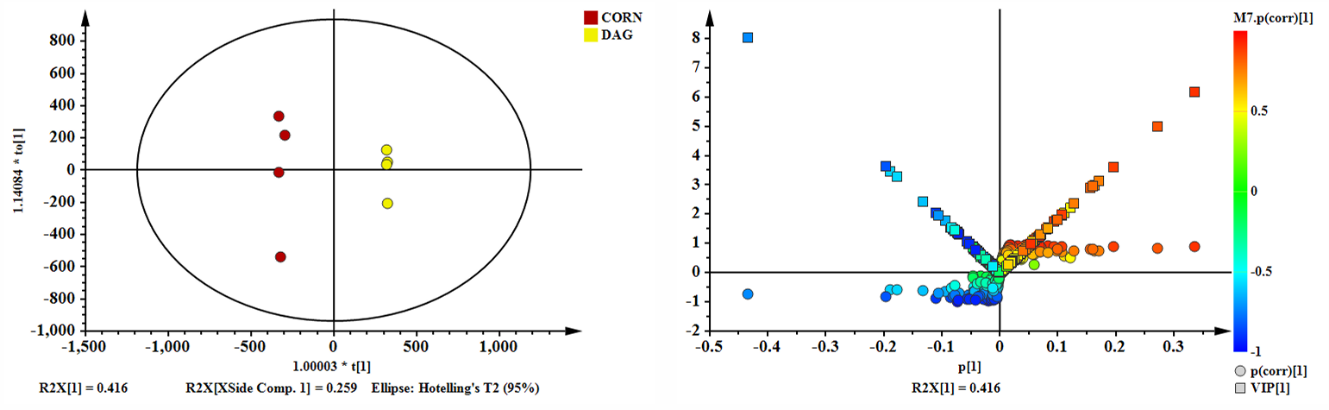


Figure S6. The OPLS - DA score plot and the loading plot based on VIP index between CORN group and DAG group
